# A folded and immunogenic IgE-hyporeactive variant of the major allergen Phl p 1 produced in *Escherichia coli*

**DOI:** 10.1186/s12896-015-0150-z

**Published:** 2015-06-09

**Authors:** Mattias Levin, Harm Otten, Claes von Wachenfeldt, Mats Ohlin

**Affiliations:** Dept. of Immunotechnology, Lund University, Medicon Village building 406, S-223 81, Lund, Sweden; Crystallization facility at the MAX IV laboratory and Lund University, Lund, Sweden; Lund Protein Production Platform (LP3), Lund University, Lund, Sweden

**Keywords:** Group 1 grass pollen allergen, Hypoallergen, IgE, Immunogenicity, Production, Protein fold

## Abstract

**Background:**

Group 1 grass pollen allergens are a major cause of allergic disease. Specific immunotherapy involving controlled administration of allergens can be used as a disease-modifying treatment for such disease. Recombinant allergen variants with reduced IgE binding capacity may be used as component in such vaccines, as they may induce fewer treatment side effects than materials currently in use. A mutated variant of the immunodominant C-terminal domain of the group 1 grass pollen allergen Phl p 1 was recently established through an approach that used a set of human monoclonal IgE as a guide to identify mutations that disturbed IgE-allergen interactions. Further analysis of this domain is required to establish its potential for use in treatment.

**Methods:**

GST-tagged wild-type and mutated C-terminal domains of Phl p 1 were produced in *Escherichia coli* TUNER(DE3). The products were purified by affinity chromatography on immobilized glutathione. GST was removed by enzymatic cleavage and tag-free products were purified by size exclusion chromatography. Products were assessed by SDS-PAGE, circular dichroism spectroscopy, differential scanning fluorimetry and dynamic light scattering. Rats were immunized with GST-tagged and tag-free mutated C-terminal domain of Phl p 1. Antigen-binding properties of induced antibodies were assessed by immunochemical analysis.

**Results:**

The mutated domain has a structure very similar to that of the wild-type domain as determined by circular dichroism, but a reduced thermal stability. Immunization of rats demonstrates that this IgE-hyporeactive domain, despite its three sequence modifications (K8A, N11A, D55A), is able to induce antibodies that substantially block the binding of allergic subjects’ IgE to the wild-type allergen.

**Conclusions:**

It is concluded that this IgE-hyporeactive molecule can be produced in folded form and that it is able to induce an antibody response that efficiently competes with IgE recognition of Phl p 1. These findings suggest that it, or a further evolved variant thereof, is a candidate for use as a component in specific immunotherapy against grass pollen allergy.

**Electronic supplementary material:**

The online version of this article (doi:10.1186/s12896-015-0150-z) contains supplementary material, which is available to authorized users.

## Background

Allergic disease substantially reduces the quality of life of affected subjects and incurs large costs to society as a whole. This disease of the immune system, is initiated by activation of effector cells carrying antibodies of the IgE isotype by their antigen, so-called allergens [1]. Allergens, often otherwise harmless compounds, thus represent one of the key components of the disease and measures that alter their ability to activate effector cells may affect allergic disease conditions.

Procedures to treat allergic disease mostly target the symptoms of disease and these procedures are not curative. The only treatment option with potential to cure allergic disease is vaccination, so-called specific immunotherapy (SIT) [2], that directly interferes with the immune reactions that cause disease. Such treatment may for instance induce allergen-specific antibodies of the IgG isotype that compete with allergy-inducing IgE for binding to allergens, thereby preventing the activation of effector cells. Such vaccination is, however, often accompanied by extensive side effects, in particular allergic reactions to the vaccine preparation that contains unmodified allergen(s). To enhance the tolerability profile of such vaccines attempts have been made to diminish the allergenicity of vaccine components while maintaining their ability to induce protective immunity. Such allergen variants, molecules that may be found in nature or may be created in vitro for instance through mutagenesis, shuffling of the order of different parts of allergens, or by selecting sequences not targeted by IgE, with diminished ability to induce allergic symptoms, are termed hypoallergens [3]. Such hypoallergens are now used for development of new vaccines to be used in SIT.

Grass pollen is a major cause of seasonal allergy in many parts of the world [4]. Consequently vaccines targeting such allergies have been established and are commercially available. These vaccines are based on natural pollen extracts, for instance from timothy (*Phleum pratense*), and they carry a risk of inducing allergy-type side effects. Pollens contain numerous allergens but the group 1 and group 5 pollen allergens are among those most commonly contributing to IgE binding [5,6]. More defined recombinant allergen vaccines, specifically incorporating some of the major allergens, including group 1 pollen allergens, of grass pollen have been created to investigate their potential as defined vaccines [7].

Group 1 pollen allergens are made up of two domains. Its C-terminal domain appears to be immunodominant in terms of induction of allergen-specific IgE in humans [8]. We recently developed an IgE-hyporeactive variant of this particular domain of the timothy group 1 allergen, Phl p 1, through the introduction of three mutations (N8A, K11A, D55A), mutations that were selected based on their ability to reduce IgE binding [9]. This knowledge-based approach depended on the utilization of allergen-specific monoclonal antibody specificities developed from the IgE repertoire of allergic subjects [8-11]. To allow further assessment of the IgE-hyporeactive domain developed this way, we now define a strategy to produce large quantities of the protein in *Escherichia coli*. We also define its ability to form a folded protein structure and its stability upon thermal stress. Finally we determine its ability to induce antibodies that also target the unmodified protein sequence, a feature important if it, or a variant of it, is to be useful in SIT.

## Results

### Recombinant production of allergen

**Figure 1:**
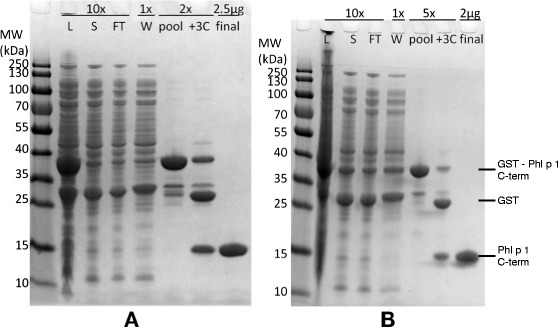
SDS-PAGE analysis of the product obtained during the different steps during the purification of the IgE hyporeactive (K8A, N11A, D55A) **(A)** and wild-type allergen **(B)** C-terminal domain of Phl p 1.0102. The following fractions were analysed: Lysate (L), soluble fraction (S), column flow through (FT) and wash (W) fractions, eluted fractions (pool), the pool after digestion with PreScission protease (+3C), and the final, purified protein (final). Dilutions of the samples prior to electrophoresis are indicated above the lanes. The purified non-GST fused IgE hyporeactive domain, depicted to the right, migrates as a single band, although at a slightly higher apparent molecular weight (14 kDa) than expected (11 kDa). The purified GST-coupled protein (37 kDa) as well as GST (26 kDa) are clearly visible with expected molecular weights.

Laboratory small-scale production of GST-allergen domain fusion protein yielded low quantities of recombinant protein [9]. To facilitate further studies of the protein and in particular to allow for production of sufficient amounts of a GST-tag-free product, the production methodology was optimized [9], as detailed in the Methods section. To have access to larger quantities of pure protein for structural studies and studies of immunogenicity, we used a purification scheme based on GST binding to immobilized glutathione, cleavage of the product with PreScission protease, and removal of uncleaved enzyme and GST by use of size exclusion chromatography. This approach provided >90% pure product of the mutated version of the C-terminal domain of Phl p 1 (Figure 1). As expected, the purified protein migrated as a single product during SDS–PAGE with an apparent molecular weight of 14 kDa protein, slightly above the expected size. The yield of purified protein was approximately 1 mg per litre of cultured bacteria.

To assess whether or not the developed processes relied on any specific features inherent only to the mutated version of the C-terminal domain of Phl p 1 we also produced and purified the module with the wild-type sequence using the same approach. These efforts yielded a product (Figure 1) with purity and in amounts similar to those found following production of the IgE-hyporeactive domain.

### Recombinant protein is monodispersed and well-folded

The state of aggregation of the purified allergen and hypoallergen was investigated with gel filtration. Both the wild-type and the mutant protein eluted with volumes corresponding to a molecular mass of approximately 20 kDa, suggesting that these proteins exist as dimers in solution (data not shown). DLS (data not shown) indicated that both protein preparations were monodisperse and contained low amounts of aggregates.

**Figure 2:**
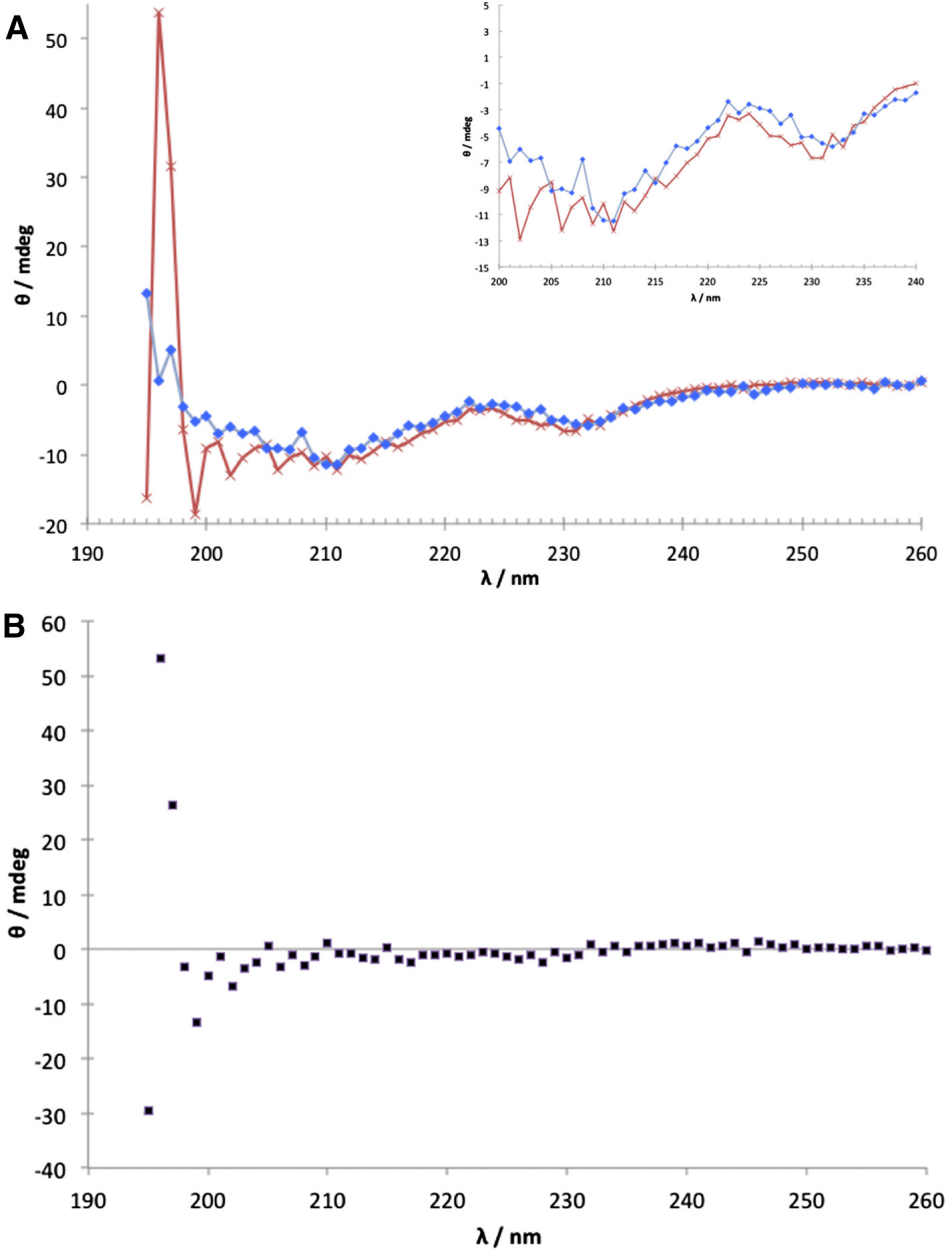
Analysis of folding by CD spectroscopy. **A**. CD spectra of instrument ellipticity output. Spectra were buffer subtracted and nulled at 260 nm. The spectra of the C-terminal domain of Phl p 1.0102 is shown in blue and its IgE-hyporeactive (K8A, N11A, D55A) variant is shown in red. Inset zooms in on the spectra between 200–240 nm. **B**. CD difference spectrum of the two proteins.

CD spectroscopy was used to estimate secondary structure content of the proteins at +4°C. Based on the similarity of the CD spectra (Figure 2) it is concluded that the content of structure of the two proteins is similar at low temperatures. In summary, there is no indication, within the accuracy and methodological limits of CD spectroscopy, that the K8A, N11A, D55A substitutions cause significant structural modifications of the protein.

**Figure 3:**
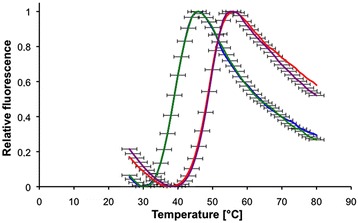
Thermal denaturation assay using Thermofluor technology with SYPRO© dye. Unfolding fluorescent curves for the C-terminal domain of Phl p 1.0102 (red, purple) and its IgE-hyporeactive sequence variant (K8A, N11A, D55A) (blue, green). A shift of 9.7°C was observed between the IgE-hyporeactive variant and the wild-type protein.

The thermal stability of the pure proteins was measured using Thermofluor technology. The calculated melting temperatures were 48.5 +/− 2.5°C for the allergen fragment and 38.8 +/− 2.5°C for the hypoallergen fragment (Figure 3). Thus the mutant protein unfolded at a temperature almost 10°C lower than the wild-type protein.

### Recombinant protein induce allergen-specific antibodies in rats

**Figure 4:**
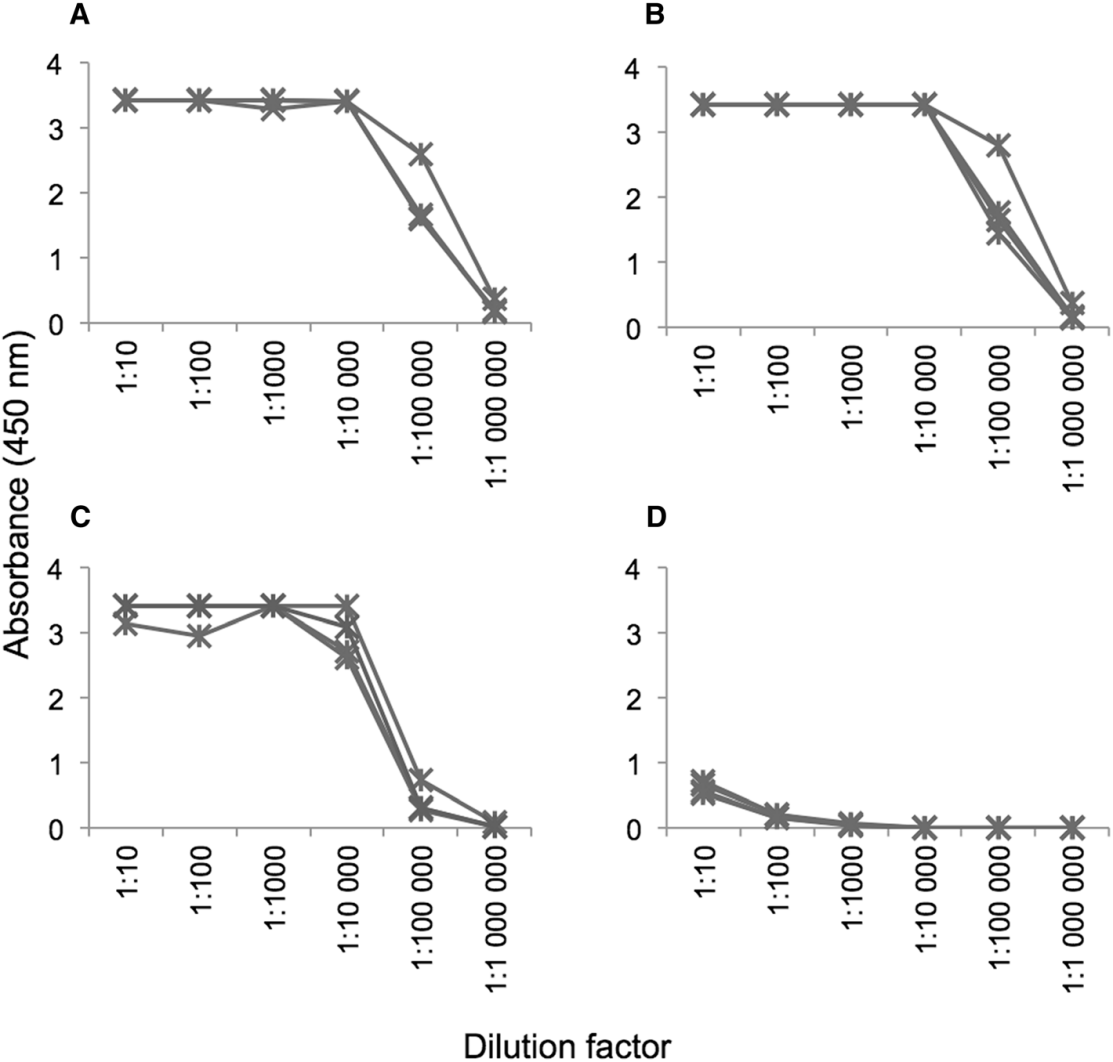
Immunoreactivity of antibodies in rat serum after immunization with the K8A, N11A, D55A mutant of the C-terminal domain of Phl p 1.0102 without the GST fusion tag **(A-C)** or PBS **(D)**. Four rats were used for each immunization. Reactivity was tested against the non-GST fused K8A, N11A, D55A mutant **(A and D)**, wild-type intact Phl p 1.0102 **(B)** and GST **(C)**. Serum antibodies obtained from rats immunized with the IgE-hyporeactive mutant C-terminal domain of Phl p 1.0102 coupled to GST showed similar reactivity to the non-GST fused K8A, N11A, D55A mutant and the intact wild-type Phl p 1.0102, while the reactivity to GST was considerably higher (data not shown).

SIT has been shown to induce antibodies of the IgG isotype with the ability to interfere with the binding of patient IgE to the allergen and it is likely beneficial to retain such an ability also in a modified hypoallergenic protein. We therefore assessed the ability of the produced IgE-hyporeactive domain to induce antibodies of the IgG isotype with specificity for the wild-type Phl p 1 allergen by immunization of rats and, if so, if these antibodies carried the desired ability to block the binding of serum IgE from allergic patients to Phl p 1. Indeed, the IgE-hyporeactive domain turned out to be immunogenic in rats and sera from rats immunized with both the GST-tag carrying recombinant protein as well as the PreScission protease-treated and purified protein contained high levels of IgG with an ability to bind both the IgE-hyporeactive domain and the wild-type Phl p 1 at comparable levels (Figure 4A, B). Notably, rats immunized with the IgE-hyporeactive protein also developed significant levels of anti-GST IgG, indicating presence of residual GST even in the PreScission protease treated and purified protein preparation, although at levels too low to be detected using standard SDS-PAGE analysis (Figure 4C). The unexpected GST-reactivity in this group of rats could potentially also be explained by cross-reactivity of IgG-responses developed against Phl p 1. To further investigate this matter inhibition assays were performed, in which the ability of both Phl p 1 and GST to block binding of rat IgG to immobilized Phl p 1 was assessed (Additional file 1: Figure S1). While Phl p 1 efficiently blocked the antigen-specific interaction between polyclonal rat IgG and the immobilized allergen, GST failed to do so even at high concentrations (10 μg/ml). These results cannot fully exclude the possibility that at small proportion of the Phl p 1-reactive rat IgG is cross-reactive to GST, or that these antibodies bind to GST with an affinity too low to allow GST in solution to block antibody binding to Phl p 1. However, it should be considered more likely that the detected GST-reactivity is due to traces of GST left in the PreScission protease treated preparation, traces that are able to induce humoral immunity to GST following immunization. Sera from control rats immunized with PBS showed no relevant reactivity with Phl p 1 (Figure 4D). Importantly, these findings demonstrate that the mutations introduced into the IgE-hyporeactive domain do not alter the protein in a way that prevent it from inducing antibody responses specific also for the wild-type Phl p 1 protein.

We then assessed the ability of the rat antibodies induced against the IgE-hyporeactive C-terminal domain of Phl p 1 to inhibit the binding of serum IgE from allergic patients to wild-type intact Phl p 1 in a competitive ELISA assay. The induced antibodies of all four immunized rats did indeed interfere (20-88% inhibition; average: 66%) with patient IgE reactivity to immobilized intact Phl p 1 (Table 1 and Additional file 1: Figure S2). In contrast, antibodies from a control rat immunized with PBS had no effect on patient IgE binding to the allergen (Additional file 1: Figure S2). Thus, despite the fact that the recombinant module used for immunization only represented a part (approximately 42% of the molecular mass) of the entire intact allergen, the antibodies it induced were able to a block a substantial part of most patients’ IgE to the intact allergen. This demonstrates the ability of the mutated IgE-hyporeactive domain to provoke antibody responses that target major IgE-reactive areas on the intact, wild-type Phl p 1 allergen, a property that is likely to be highly beneficial in SIT of allergic disease. As the IgE of a few patients (Table 1) was not efficiently blocked from binding Phl p 1, it is possible that the immune response of some subjects is not so focused on the otherwise immunodominant [8] C-terminal domain of Phl p 1. Further studies will be required to address this matter in larger patient cohorts.

**Table 1:** **Inhibition of binding of patients’ serum IgE (diluted 1:25) to intact Phl p 1 by polyclonal rat antibodies (diluted 1:1000) induced by immunization with the IgE-hyporeactive domain without the GST fusion tag**

	Inhibition of serum IgE binding* (%)				
**Patient**	**Rat 1**	**Rat 2**	**Rat 3**	**Rat 4**	**Mean ± SD (%)**
1	83	85	84	78	83 ± 3
2	25	42	37	20	31 ± 10
3	83	74	79	71	77 ± 5
4	49	51	50	39	47 ± 6
5	67	65	51	52	59 ± 8
6	84	81	81	80	81 ± 2
7	88	85	85	87	86 ± 2
8	60	55	73	73	65 ± 9

*As compared to reactivity in the presence of sera from control rat immunized with PBS.

## Discussion

IgE-hyporactive proteins with potential to induce antibodies that prevent IgE binding to wild-type allergen are candidates for development of improved vaccines for use in SIT against allergy. We recently defined a mutated variant of the immunodominant domain of Phl p 1, a major grass pollen allergen, with substantially reduced ability to bind Phl p 1-specific IgE [9]. To allow further studies of this module and to facilitate a decision-making process that will define its usefulness, and any potential need for further molecular adaptation, we used a production methodology [9] that allowed us to obtain mg quantities of protein for further analysis. By use of the *E. coli* TUNER(DE3) strain, that allows more controlled and even uptake of the inducer (IPTG) of protein production, reduction of the production temperature, and extension of the production time we were able to obtain multi-mg quantities of the fusion protein product for further detailed studies of its properties. Elimination of the N-terminal GST fusion tag was achieved using PreScission protease treatment and subsequent chromatorgraphy, as determined by gel electrophoresis. Although trace amounts of GST appear to remain in the purified, PreScission protease treated, tag-free protein preparation, the procedure was shown to provide a well-folded domain for use in applications that require several mg of product.

IgE antibodies are believed to mostly recognize conformational epitopes that depend on an intact protein structure [12]. Indeed, the two determined crystal structures of human IgE fragments in complex with their respective allergens illustrate this aspect of IgE-allergen interactions [13,14]. Consequently, strategies to develop hypoallergenic molecules in some respects often rely on destruction of protein fold [15,16]. This particular IgE-hyporeactive module [9], however, retain its folded character, which is undistinguishable from that of the wildtype domain as determined by CD. The approach [9] whereby it was created may in part be responsible for this feature. This process relied on identification of multiple surface-exposed residues that are directly responsible for monoclonal human IgE binding that also translate into reduced binding to polyclonal IgE [9]. Importantly, amino acid side chains that are involved in establishing the core of the protein fold are largely not affected by the mutations. We consequently believe that such a minimal, knowledge-based, approach is more likely than a more extensive mutagenesis approach to minimally affect protein structure and it will leave much of the surface that is not critical for IgE binding untouched. The process thus has the potential to induce a wide range of antibodies that also target the natural allergen, the purpose of an SIT strategy.

Although the hypoallergen was folded at +4°C it was more sensitive to thermal denaturation and melted *in vitro* at a temperature close to normal body temperature. The precise molecular effect responsible for this destabilization towards heating is not known. One of the modifications of the IgE-hyporeactive variants (N11A), however, affects a residue whose side chain has the potential to establish two polar interactions, more specifically with protein backbone amino groups of residues N13 and Y14 (Additional file 1: Figure S3). It is conceivable that this mutation at least in part contributes to the thermal destabilization of the IgE-hyporeactive properties of the mutant protein but further investigations are required to assess the precise role of the these modifications in molecular destabilization. Nevertheless, despite the fact that mutant module is more sensitive towards thermal denaturation, it has been possible to establish a production and purification procedure that produces a well-folded protein.

## Conclusions

In conclusion, we herein demonstrate features of a folded, immunogenic, recombinant IgE-hyporeactive domain of the major grass pollen allergen Phl p 1, a domain that has been developed through a knowledge-based approach [9]. It is suggested that this domain, variants of it, or ultimately other IgE-hyporeactive domains established by this methodology, after extensive assessment of safety and efficacy, has the potential to find application in safer allergy vaccination.

## Methods

### Production and purification of recombinant protein

Codon-optimised genes (Additional file 1: Figure S4) encoding the C-terminal domain of Phl p 1.0102 or a mutant (K8A, N11A, D55A; residues are numbered starting with the KVTF sequence in the beginning of the C-terminal domain) thereof with reduced IgE-binding capacity have been described in the past [9]. These genes were cloned into the pGEX-6P1-vector (GE Healthcare, Uppsala, Sweden) and originally used for production of recombinant protein in *E. coli* T7 express (New England Biolabs, Ipswich, MA). Improved yield [9] was obtained using strain *E. coli* TUNER (DE3) (F^−^*ompT hsdSB* (r_B_^−^ m_B_^−^) *gal dcm lacY1*(DE3)) (Novagen). This strain is derived from E. coli BL21 and lacks the lacY gene encoding the Lac permease. In contrast to the previously used strain [9] the T7 RNA polymerase is driven from the lacUV5 promoter and present in a lambda prophage. Briefly, *E. coli* TUNER(DE3) was transformed with either plasmids encoding the C-terminal domain of Phl p 1.0102 or the mutant thereof. The cells were grown in of LB broth (Difco) supplemented with 100 μg/ml ampicillin in 5 L Erlenmeyer flasks with indentations (1 L/flask) at 18°C, 200 rpm. At OD_600_ ~ 0.5, IPTG was added to a final concentration of 1 mM. 18 hours after induction, cells were harvested in a JLA 8.1000 rotor, 8000×g, 4°C, 15 min, and the pellets were stored at −80°C or used directly for preparation of soluble extract. The use of a low production temperature (18°C) and an extended production phase (18 h) were important parameters to reach a high product yield. The pellets from 1 L culture were resuspended in 20 ml PBS, pH 7.3, supplemented with one tablet Complete Protease Inhibitor, EDTA-free (Roche). The cell suspension was passed twice through a French Pressure cell at 18 000 psi. The lysate was centrifuged in a Beckman Ti 50.2 rotor, 45 000 rpm, 60 min, 4°C, and the supernatant (soluble fraction) was collected and passed through a 0.45 μm filter.

The soluble fraction was used for affinity chromatography. A 5 ml GSTrap FF column (GE Healthcare) was connected to an ÄKTA Avant system (GE Healthcare). The column was run at room temperature, while fractions were collected at 4°C. The column was washed with PBS, pH 7.3 until a stable signal at 280 nm was obtained. Bound protein was eluted with 10 column volumes 50 mM Tris–HCl, 150 mM NaCl, 40 mM reduced glutathione (pH 8.0). Peak fractions were analyzed with SDS-PAGE, and fractions containing allergen were pooled. The pooled fractions were concentrated and the buffer was exchanged to 50 mM Tris, 150 mM NaCl, 1 mM EDTA (pH 8.0) using Vivaspin Turbo 15 ultrafiltration spin columns (molecular weight cutoff: 10,000 Da). The glutathione S-transferase (GST)-tag was removed with PreScission protease (13 u/mg protein) (GE Healthcare). DTT was added to the reaction mixture to a final concentration of 1 mM and the cleavage reaction was left at 4°C for one day. To separate allergen from PreScission protease and GST and other contaminating proteins, size exclusion chromatography was performed using a HiLoad 26/600 Superdex 200 pg gel filtration column (GE Healthcare). The column was run at 4°C, with a flow rate of 2.6 ml/min 50 mM Tris, 150 mM NaCl, pH 8.0, and 4 ml fractions were collected. Peak fractions were analyzed with SDS-PAGE, and fractions containing allergen were pooled. The pooled fractions were concentrated and the buffer was exchanged to PBS using Vivaspin Turbo 15 ultrafiltration spin columns (molecular weight cutoff: 3,000 Da). The concentrated protein solution was passed through a 0.22 μm sterile filter and was stored at +4°C.

GST, not coupled to allergen, was produced in *E. coli* T7 express (New England Biolabs) using the pGEX-6P-1 vector (GE Healthcare) without any gene insert, and purified using GSTrap FF columns (GE Healthcare), as previously described [9].

### Immunization of rats with IgE-hyporeactive protein

Rats were immunized four times with either (1) recombinant C-terminal domain carrying the K8A, N11A, D55A modifications fused to GST, or (2) a GST-free variant thereof purified following PreScission protease treatment (approximately 200 (primary immunization) or 100 (booster immunization) μg protein was used/injection), or (3) PBS, in combination with Freund’s adjuvant (complete adjuvant was used for primary immunization while incomplete adjuvant was used for booster immunization) (Agrisera, Vännäs, Sweden). The procedure was approved by the Ethical Committee on Animal Experiments in Umeå, Sweden.

### Determination of specific IgG levels in rat serum, specific IgE levels in patient serum and ability to inhibit patient IgE binding to Phl p 1

To determine the levels of specific IgG in serum from immunized rats or specific IgE levels in serum from allergic donors, recombinant Phl p 1.0102 (Biomay, Vienna, Austria), the IgE-hyporeactive mutant C-terminal domain thereof or GST were coated into microtiter plates (Corning, Corning, NY) at a concentration of 1 μg/ml or 0.2 μg/ml in PBS. After washing with 0.9% (w/v) NaCl and 0.05% (v/v) Tween 20, rat or patient sera, diluted to suitable concentrations in blocking buffer (1% (w/v) non-fat dry milk and 0.05% (v/v) Tween 20 in PBS), was added and incubated for 1 h at 37°C. After an additional wash step bound rat IgG was detected using a horse-radish peroxidase-labelled goat anti-rat IgG antiserum (Bethyl Laboratories, Montgomery, TX) using 1-Step Ultra TM-ELISA substrate (Thermo Scientific, Rockford, IL) as chromogen. Absorbance was measured at 450 nm. Bound patient IgE was detected using a horse-radish peroxidase-labelled anti-IgE antiserum (KPL, Guilford, UK) and chemiluminescent substrate (SuperSignal ELISA Femto Maximum Sensitivity Substrate, Thermo Scientific).

The ability of serum from immunized rats to inhibit patient IgE reactivity to Phl p 1 was assessed using a competitive ELISA assay. Sera had been obtained following informed consent from allergic subjects, as approved by the Regional Ethical Committee at Lund University. Microtiter plates were coated with Phl p 1.0102 at a concentration of 0.2 μg/ml in PBS and washed as described above. Next, serum from rats immunized with either the IgE-hyporeactive domain, not coupled to GST, or PBS, diluted 1:1000 in blocking buffer or PBS as reference, was added and incubated for 1 h at 37°C. Without washing, serum from allergic patients were added to the wells pre-incubated with rat serum or PBS at a final 1:25 dilution and incubated for 1 h at 37°C. After washing, bound IgE was detected using a horse-radish peroxidase-labelled anti-IgE antiserum and chemiluminescent substrate. The degree of inhibition was assigned by relating the signal obtained from the wells pre-incubated with rat serum to the well pre-incubated with PBS.

### Circular dichroism spectroscopy

Circular dichroism (CD) spectroscopy measurements were performed on a Jasco J-810 spectropolarimeter equipped with a Jasco CDF-426S Peltier set to 4°C. The protein concentration used was 0.6 mg/ml and averages of five scans were baseline-subtracted (buffer: 100 mM ammonium phosphate, 10 mM potassium phosphate, pH 7.3).

### Thermal stability assay

To evaluate the effects of amino acid substitutions on the overall thermal stability for wild type and mutant allergen, differential scanning fluorimetry (DSF) assays were performed using a Mx3005P qPCR system (Stratagene). The protein concentration was 120 μM in 100 mM sodium sulphate, 20 mM potassium phosphate, pH7.3, containing 5 × SYPRO orange dye (1:1000 dilution) (Invitrogen). The fluorescence was measured as a function of increasing temperature at the rate of 1°C/min (excitation wavelength: 498 nm; emission wavelength: 610 nm).

### Dynamic Light Scattering (DLS) measurements

DLS measurements were performed at 10°C using a Malvern Zetasizer DLS instrument. Data analysis was done using the Zetasizer software supplied with the instrument. Protein (0.6 mg/ml) in buffer 100 mM ammonium phosphate, 10 mM potassium phosphate, pH 7.3 was used for the analysis.

## Additional file

Additional file 1:
**Supplementary Information.**

## Abbreviations

CD: Circular dichroism
DLS: Dynamic light scattering
DSF: Differential scanning fluorimetry
GST: Glutathione S-transferase
SIT: Specific immunotherapy

## References

[1] Gould HJ, Sutton BJ (2008). IgE in allergy and asthma today. Nat Rev Immunol.

[2] Linhart B, Valenta R (2012). Vaccines for allergy. Curr Opin Immunol.

[3] Valenta R, Linhart B, Swoboda I, Niederberger V (2011). Recombinant allergens for allergen-specific immunotherapy: 10 years anniversary of immunotherapy with recombinant allergens. Allergy.

[4] Bauchau V, Durham SR (2004). Prevalence and rate of diagnosis of allergic rhinitis in Europe. Eur Respir J.

[5] Laffer S, Vrtala S, Duchêne M, van Ree R, Kraft D, Scheiner O (1994). IgE-binding capacity of recombinant timothy grass (Phleum pratense) pollen allergens. J Allergy Clin Immunol.

[6] Niederberger V, Laffer S, Fröschl R, Kraft D, Rumpold H, Kapiotis S (1998). IgE antibodies to recombinant pollen allergens (Phl p 1, Phl p 2, Phl p 5, and Bet v 2) account for a high percentage of grass pollen-specific IgE. J Allergy Clin Immunol.

[7] Jutel M, Jaeger L, Suck R, Meyer H, Fiebig H, Cromwell O (2005). Allergen-specific immunotherapy with recombinant grass pollen allergens. J Allergy Clin Immunol.

[8] Flicker S, Steinberger P, Ball T, Krauth MT, Verdino P, Valent P (2006). Spatial clustering of the IgE epitopes on the major timothy grass pollen allergen Phl p 1: importance for allergenic activity. J Allergy Clin Immunol.

[9] Levin M, Rydnert F, Källström E, Tan LW, Wormald PJ, Lindstedt M (2013). Phl p 1-specific human monoclonal IgE and design of a hypoallergenic group 1 grass pollen allergen fragment. J Immunol.

[10] Gadermaier E, Levin M, Flicker S, Ohlin M (2014). The human IgE repertoire. Int Arch Allergy Immunol.

[11] Persson H, Karbalaei Sadegh M, Greiff L, Ohlin M (2007). Delineating the specificity of an IgE-encoding transcriptome. J Allergy Clin Immunol.

[12] Aalberse RC, Crameri R (2011). IgE-binding epitopes: a reappraisal. Allergy.

[13] Niemi M, Jylhä S, Laukkanen ML, Söderlund H, Mäkinen-Kiljunen S, Kallio JM (2007). Molecular interactions between a recombinant IgE antibody and the beta-lactoglobulin allergen. Structure.

[14] Padavattan S, Flicker S, Schirmer T, Madritsch C, Randow S, Reese G (2009). High-affinity IgE recognition of a conformational epitope of the major respiratory allergen Phl p 2 as revealed by X-ray crystallography. J Immunol.

[15] Linhart B, Valenta R (2012). Mechanisms underlying allergy vaccination with recombinant hypoallergenic allergen derivatives. Vaccine.

[16] Vrtala S, Focke-Tejkl M, Swoboda I, Kraft D, Valenta R (2004). Strategies for converting allergens into hypoallergenic vaccine candidates. Methods.

