# Supplementary material for: A folded and immunogenic IgE-hyporeactive variant of the major allergen Phl p 1 produced in Escherichia coli
Source: BMC Biotechnol. 2015 Jun 9;15:52. doi: 10.1186/s12896-015-0150-z (PMC4460866; doi:10.1186/s12896-015-0150-z)
Supplement: Additional file 1: — Supplementary Information. [file 12896_2015_150_MOESM1_ESM.pdf]

SUPPLEMENTARY INFORMATION

A folded and immunogenic IgE-hyporeactive variant of the major allergen Phl p 1 produced in *Escherichia coli*

Mattias Levin[1], Harm Otten[2, 3], Claes von Wachenfeldt[3], Mats Ohlin[1,*]

[1] Dept. of Immunotechnology, Lund University, Lund, Sweden

[2] Crystallization facility at the MAX IV laboratory and Lund University, Lund, Sweden.

[3] Lund Protein Production Platform (LP3), Lund University, Lund, Sweden.

* **Corresponding author:** Dr. Mats Ohlin, Dept. of Immunotechnology, Medicon Village building 406, S-223 81 Lund, Sweden; E-mail: mats.ohlin@immun.lth.se; Telephone: +46-46-2224322; Telefax: +46-46-2224200.

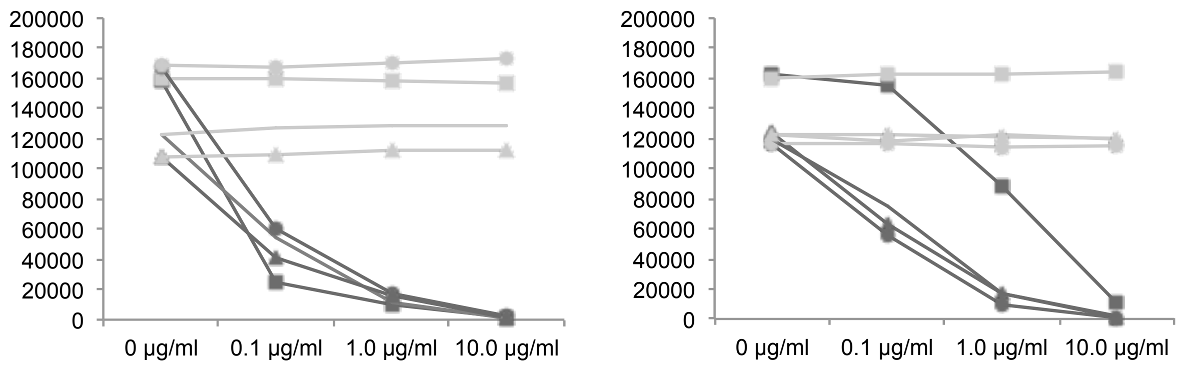

Supplementary Figure 1. Inhibition of the binding of rat sera to immobilized Phl p 1.0102, coated at 0.2µg/ml, by Phl p 1.0102 (dark grey) and GST (light grey), as determined using ELISA. Both sera from rats immunized with the non-GST tagged mutant version of the C-terminal domain of Phl p 1.0102 (left chart; rat 1, circles; rat 2, boxes; rat 3, triangles; rat 4, no marker) and GST-tagged protein (right chart; rat 5, circles; rat 6, boxes; rat 7, triangles; rat 8 no marker) were efficiently inhibited by Phl p 1.0102, while no inhibition was detectable using GST, indicating a Phl p 1-specific response.

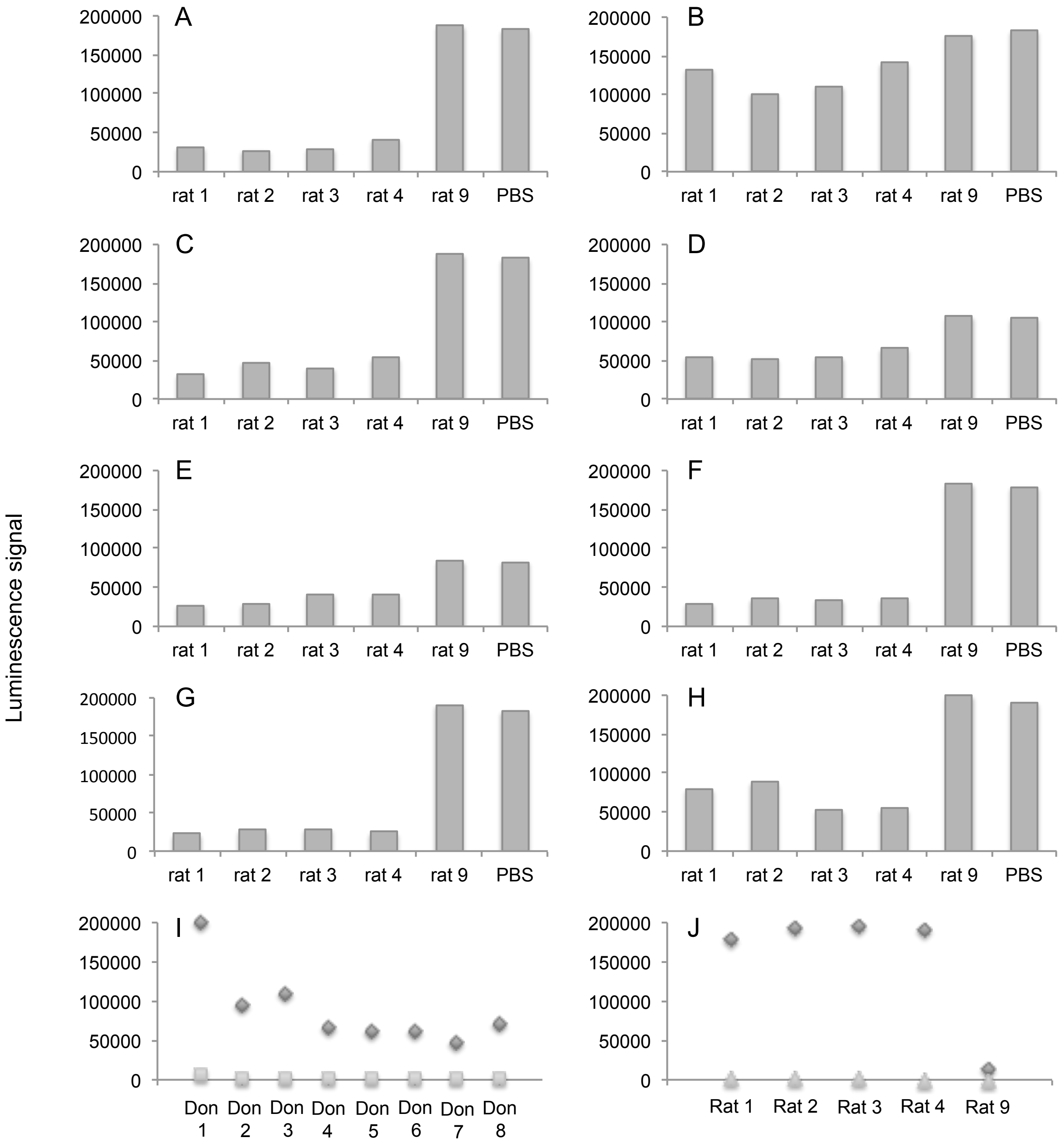

Supplementary Figure 2. Inhibition of binding of IgE in eight patient sera to wild-type Phl p 1.0102 (A-H) by serum from rats immunized with the non-GST tagged IgE hyporeactive (K8A, N11A, D55A) mutant C-terminal domain of Phl p 1.0102 (rat 1-4), serum from control rat immunized with PBS (rat 9) and PBS, as determined using ELISA. All investigated donor sera showed IgE binding to Phl p 1.0102 coated at 0.2 µg/ml at levels well above background binding to GST (I) and serum IgG from rats 1-4 showed

similarly good binding to Phl p 1.0102 coated at this level (J), while rat 9, immunized with PBS, showed minimal binding to Phl p 1.0102, as compared to background binding to BSA.

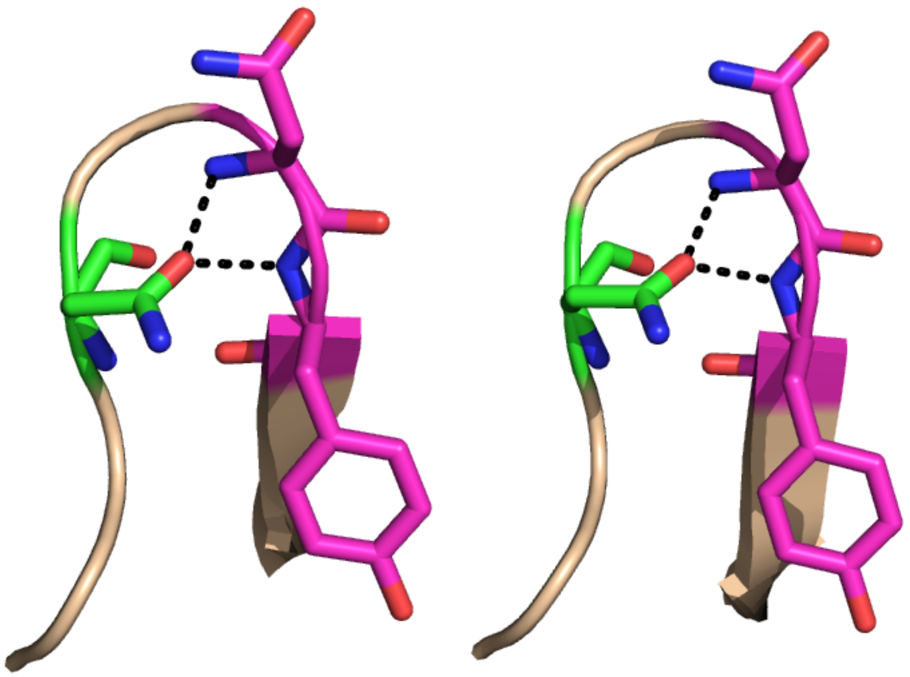

Supplementary Figure 3. Proposed polar interactions made by the side chain of residue N11 (green), a residue mutated in the IgE hyporeactive variant of the C-terminal domain of Phl p 1, to protein backbone atoms in the two molecules present in the unit cell of the Phl p 1 structure (PDB: 1N10).

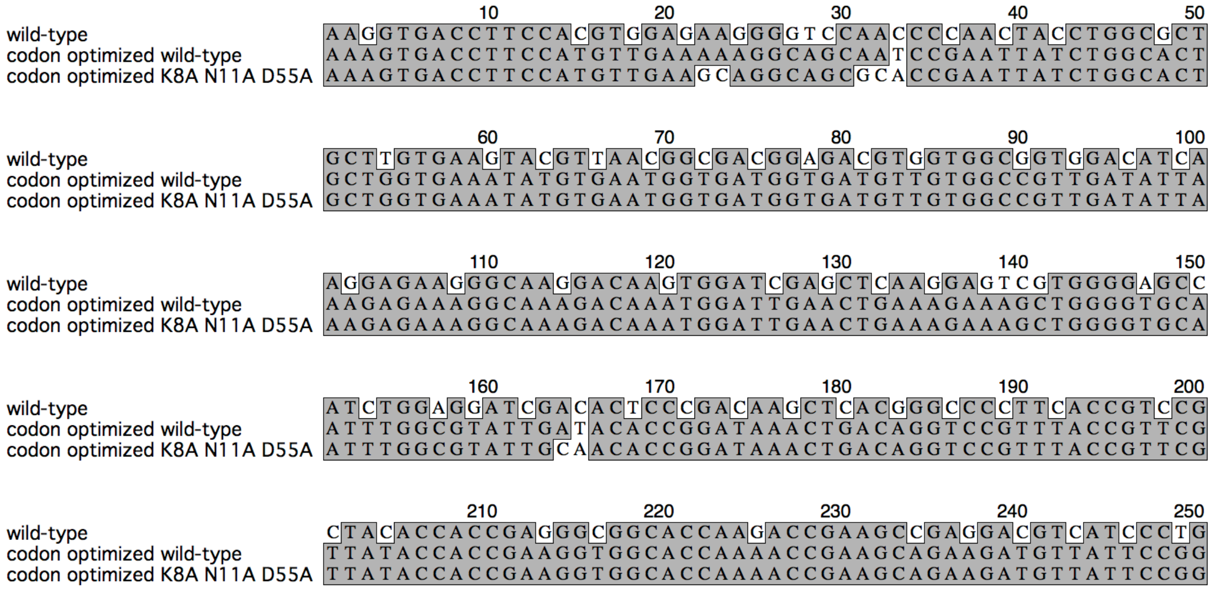

Supplementary Figure 4. Sequences of the part of the wild-type gene (GenBank: X78813) that encodes the C-terminal domain of Phl p 1.0102 and the codon-optimized sequences that encode the same wild-type domain and the N8A, K11A, D55A mutant thereof.
